# Interaction of biomedical nanoparticles with the pulmonary immune system

**DOI:** 10.1186/s12951-016-0242-5

**Published:** 2017-01-09

**Authors:** Fabian Blank, Kleanthis Fytianos, Emilie Seydoux, Laura Rodriguez-Lorenzo, Alke Petri-Fink, Christophe von Garnier, Barbara Rothen-Rutishauser

**Affiliations:** 1 Respiratory Medicine, Bern University Hospital, University of Bern, Murtenstrasse 50, 3008 Bern, Switzerland; 2 Adolphe Merkle Institute, University of Fribourg, Fribourg, Switzerland; 3 Chemistry Department, University of Fribourg, Fribourg, Switzerland

**Keywords:** Biomedical nanoparticles, Immune-modulation, Specific targeting, Pulmonary antigen presenting cells, In vivo models

## Abstract

Engineered nanoparticles (NPs) offer site-specific delivery, deposition and cellular uptake due to their unique physicochemical properties and were shown to modulate immune responses. The respiratory tract with its vast surface area is an attractive target organ for innovative immunomodulatory therapeutic applications by pulmonary administration of such NPs, enabling interactions with resident antigen-presenting cells (APCs), such as dendritic cells and macrophages. Depending on the respiratory tract compartment, e.g. conducting airways, lung parenchyma, or lung draining lymph nodes, APCs extensively vary in their number, morphology, phenotype, and function. Unique characteristics and plasticity render APC populations ideal targets for inhaled specific immunomodulators. Modulation of immune responses may operate in different steps of the immune cell-antigen interaction, i.e. antigen uptake, trafficking, processing, and presentation to T cells. Meticulous analysis of the immunomodulatory potential, as well as pharmacologic and biocompatibility testing of inhalable NPs is required to develop novel strategies for the treatment of respiratory disorders such as allergic asthma. The safe-by-design and characterization of such NPs requires well coordinated interdisciplinary research uniting engineers, chemists biologists and respiratory physicians. In this review we will focus on in vivo data available to facilitate the design of nanocarrier-based strategies using NPs to modulate pulmonary immune responses.

## Background

The human respiratory tract provides a vast epithelial surface area for air conduction and gas-exchange with a combined surface area that is about 150 m^2^. In particular, the gas exchange region provides the major part of surface, where the structural barrier between air and blood is reduced to a mean arithmetic thickness of 2.2 μm or thinner tissue layers in the alveoli [1]. The vast surface and direct contact with environment makes lung the most important portal of entry for inhaled xeniobiotics such as particulate matter (reviewed in [2]). This has raised concerns that particles may cause respiratory disease or trigger adverse effects as seen with ambient combustion-derived particles recognized as an important cause of cardiovascular morbidity and mortality in areas with air pollution [3–5]. On the other hand, the unique lung characteristics render this organ ideal for novel biomedical applications by inhalation of specifically designed nanomaterials [6]. Nano-sized carriers [e.g. mainly nanoparticles (NPs) with all three dimensions below 100 nm (ISO/TS, 2008)] have been proposed as promising novel diagnostic, therapeutic, and vaccination approaches for a variety of human diseases [7–9].

Drug delivery through the pulmonary route offers several advantages over oral or parenteral delivery. This is primarily due to the presence of a dense vasculature, the circumvention of the first pass effect, and a lower concentration of drug-metabolizing enzymes in the lung combined with the highly dispersed nature of an aerosol [10, 11]. Furthermore, size-dependent deposition and size-dependent uptake by specific immune cell subsets (as discussed later) in the pulmonary compartment may lead to modulation specific downstream immune responses with reduced side-effects due to targeted delivery by NPs. Novel NPs may be employed to deliver drugs or may act as immunomodulators, either on the entire lung surface or by targeting a particular cell population localized in a specific compartment of the respiratory tract. Knowledge about the anatomical compartments in the respiratory tract and resident cells is a prerequisite to understand the interplay between APCs and inhalable NPs. In addition, each NP type requires thorough characterization and testing in vitro, before being considered for animal experimentation and clinical applications. Characterization begins during and immediately after synthesis of NPs to monitor physicochemical properties, size, shape and stability. In a subsequent step, cell-free assays can be employed to investigate how particles interact with constituents of biological solutions, such as free proteins and enzymes [12, 13]. Mechanisms of particle-cell interaction and cytotoxicity are investigated by in vitro experiments using either cell mono-cultures or more advanced and complex 3D co-culture systems that simulate specific human organs or organ compartments [13]. To study effects of NPs on the entire organism, in vivo animal models are necessary in species that represent appropriate models for the human anatomy, physiology, and immunology as closely as possible. Extensive short, intermediate and longterm in vivo characterization of both unwanted biological effects and efficacity of particle are a prerequisite before clinical testing can be performed. Such a cascade of characterization of biocompatibility and immunogenicity on multiple levels of increasing complexity will allow the development of NPs of acceptable safety and accurately defined effects regarding targeting, interplay with target cells/tissues and persistence. In particular safety regarding toxicological and immunomodulatory effects of newly developed biomedical NPs should be of major concern.

In this review we will summarize the anatomy of the respiratory tract regarding the different immune cell subsets which are populating its diverse compartments. Furthermore, we will focus on recently emerged in vivo models to monitor the immunomodulatory potential of biomedical NPs while discussing characteristics of potential biomedical NPs, which are important in order to modulate immune responses in the lung.

## General anatomy of the respiratory tract

As previously outlined, the lung provides an attractive portal of entry in the human body for non-invasive applications using biomedical NPs. Detailed knowledge on the macroscopic structure of the lung anatomy, i.e. different compartments; and in particular the distribution and function of immune cells within different compartments of the respiratory tract is crucial to develop and engineer specific inhalable NPs. The human respiratory tract is structurally designed for gas exchange in the human body through a huge internal surface area of about 150 m^2^ (i.e. alveoli and airways) closely enmeshed with a dense capillary network [1]. The respiratory tract is anatomically subdivided into four regions: (1) the extra thoracic region comprising the anterior nose and the posterior nasal passages, larynx, pharynx and mouth; (2) the bronchial region consisting of the trachea and bronchi; (3) the bronchiolar region consisting of bronchioles and terminal bronchioles; and finally (4) the alveolar-interstitial region consisting of respiratory bronchioles (bronchioles with some alveoli apposed), the alveolar ducts and sacs with their alveoli and the interstitial connective tissue, inside the interalveolar septa.

The epithelial tissue changes its architectural and cellular characteristics from the upper airway to the periphery. Beginning at the trachea/bronchi, the airway epithelium is pseudostratified with ciliated epithelial cells, i.e. mucocilary escalator, and at the level of smaller bronchioles it is of cuboidal appearance. Toward the lung periphery, the alveoli are lined by squamous cells, the alveolar type I epithelial cells which cover about 95% of the surface and share a basement membrane with the endothelial cells covering the pulmonary capillaries, and also contain alveolar type II epithelial cells, which secrete lung surfactant (surface active agent) to prevent alveolar collapse [14, 15]. The structural barrier between air and blood is reduced to a mean arithmetic thickness of 2.2 μm or thinner tissue layers in the alveoli [1]. More than 40 different cell types, amongst others different types of epithelial cells, endothelial cells, fibroblasts, nerve cells, lymphoid cells, gland cells, dendritic cells and macrophages, add to the complexity of the epithelium in the lung. All four regions in the respiratory tract contain lymphatic tissue or specific components of it [14].

There are approximately 400 million alveoli in the lungs [16], with a combined surface area that is about 140 m^2^ and with an alveolar epithelium which can be as thin as 0.1 μm [1, 15]. The interstitium of the alveolar septum is for most parts extremely thin and endothelial cells, which cover the inner surface of the capillaries, fuse with basement membranes of epithelial cells to minimize the air-blood barrier. At the thicker parts, where the basement membranes of endothelial and epithelial cells are separated, elastic fibers, collagen fibrils bundles as well as fibroblasts are present in the extracellular matrix. This large surface area, combined with an extremely thin barrier between the pulmonary lumen and the capillaries, creates conditions that are well suited for efficient gas transfer [14].

## Lung barriers and particle clearance

A series of structural and functional barriers protect the respiratory system against both harmful and innocuous xenobiotics [17]. The airway mucosa, with its respiratory epithelium sealed by apically localized tight junction complexes, provides a mechanical barrier that protects against effects of inhaled and on the lung cell surface deposited xenobiotics. Furthermore, ciliated epithelial cells and mucus producing goblet cells, together with locally produced secreted immunoglobulins (mainly IgA), provide effective mechanisms for mucociliary clearance of inhaled particulate antigens [18]. In addition, airway epithelial cells have key roles in the regulation of lung homeostasis by secretion of a range of regulatory and effector molecules (e.g. mucins, surfactant proteins, complement and complement cleavage products, antimicrobial peptides) that are involved in front-line defence against pathogens [19].

The clearance kinetics in the lung periphery is much slower due to the absence of mucociliary action, and particles are eliminated by (1) phagocytosis with subsequent transport by macrophages, (2) dendritic cells with trafficking to draining lymph nodes, as well as (3) direct translocation via the air-blood tissue barrier into the circulation. All these mechanisms by which the particles are eliminated from the inner surface of the respiratory tract have to be taken into account for the design of new NPs [20].

## The immune system in the respiratory tract

**Fig. 1:**
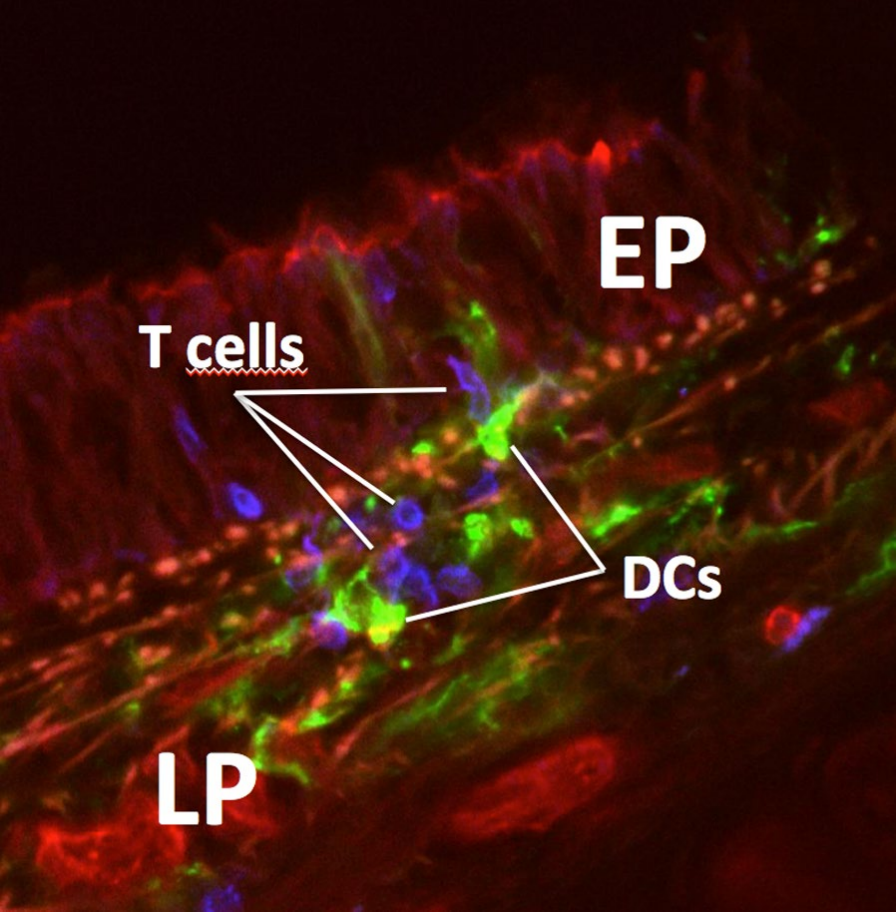
Interactions of DCs and T cells in the airway mucosa visualized by laser scanning microscopy. Micrograph shows a scanned area from a cross section through a trachea (rat). T cells (CD3, *blue*) are visible closely interacting with DCs (MHC class II, *green*) inside the airway epithelium (EP) and the lamina propria (LP)

APCs such as alveolar and interstitial macrophages, as well as dendritic cells (DCs) (Fig. 1), play an important role in the regulation of the immune response.

Respiratory tract macrophages play an important role in the maintenance of immunological homeostasis and host defense. In the lungs the key population is composed of alveolar macrophages. Under steady state conditions, the most important function of alveolar macrophages is phagocytosis and sequestration of antigen from the immune system to shield local tissues from the development of specific immune responses [21]. Alveolar macrophages have been shown to take up most of the particulate material that is delivered intranasally [22]. Since alveolar macrophages do not migrate to the lung draining lymph nodes [23], their antigen presentation capabilites are limited to interact with local effector T cells only, in contrast to pulmonary dendritic cells which, as professional antigen presenting cells, migrate to the lymph nodes in order to activate naive T cells and to direct their differentiation into effector T cells, as described later. Besides clearance of inhaled particulates, macrophages are involved in diverse functions that are achieved by the plasticity of these cells that, depending on signals present in their microenvironment, can polarize into a plethora of different phenotypes [24, 25]. Cytokines, such as interferon (IFN)-γ and tumor necrosis factor (TNF)-α, or bacterial products, such as lipopolysaccharide (LPS), induce polarization of macrophages into a proinflammatory phenotype through the transcription factor IFN regulatory factor 5 (IRF5). Such macrophages were conventionally named M1-dominant macrophages and release proinflammatory cytokines interleukin (IL)-12, IL-1β, and TNF-α. They are thus important in host defense against intracellular pathogens [24, 26]. Furthermore, macrophages induced by the proallergic asthma cytokines IL-4 and IL-13, in the past conventionally also known as M2 macrophages that are important in wound healing and host defense against helminth infections. This macrophage subset is characterized by upregulation of the mannose receptor (CD206) and, in mice, production of the chitinase-like protein YM1. However, recent literature has challenged the existence of an M1 and M2 paradigm of macrophage activation and proposed a more complex system of macrophage polarization [27]. Another macrophage phenotype consists of anti-inflammatory macrophages that are induced by compounds and mediators such as corticosteroids, IL-10, or prostaglandin E_2_ PGE_2_. Such anti-inflammatory macrophages are also characterized by upregulation of CD206, but produce the anti-inflammatory cytokine IL-10 [28].

The mucosa of the airways and the lung parenchyma also contains dense networks of DCs that develop early in life [29]. DCs are professional APCs that link innate and adaptive immunity, and therefore occupy a key role in regulating the body’s immune responses [30]. They are strategically positioned for antigen uptake both within and directly below the surface epithelium and extend protrusions into the airway lumen [31], or the alveolar space [32] similar to what has been demonstrated for DCs in intestinal tissue where DCs have been shown to form tight junction complexes with epithelial cells [33]. This characteristic suggests that DCs can sample directly, both from the airway lumen and the alveolar space [32] through the intact epithelium [31] by the expression of adherens and tight junction proteins which might help to preserve the epithelial integrity in a trans-epithelial network [34]. Morphologically characterized by dendrite-like projections DCs are the most potent APC population able to provide T cell activation (Fig. 1) [35].

Potential pathogens are ‘sensed’ through pattern recognition receptors (PRRs) that interact with pathogen associated molecular patterns (PAMPs), triggering innate and adaptive immunity [36]. In DCs, activation through the PRRs leads to upregulation of the chemokine receptor CCR7 (CD197; the ligand is CCL19/ECL) that is essential for DC migration from the site of pathogen encounter to lymph nodes, where activation of naive T cells occurs. In this process of trafficking to the lymph nodes DCs differentiate from a so-called ‘immature’ state (high capacity for antigen uptake, low capacity for T cell activation) to a ‘mature’ state (low capacity for antigen uptake, high capacity for T-cell activation) [37]. Following migration to lymph nodes, DCs face their most important task: that is to instruct T cells to respond to presented antigen in the most appropriate way. The type and activation state of the DC, the dose of antigen, as well as the nature of concomitant micro-environmental factors present at the time of antigen encounter determine the nature of the resulting T cell response [37]. Conventionally, three different outcomes for effector T cells have been distinguished: T helper 1 (Th1), T helper 2 (Th2) and regulatory T cells (Treg). A Th1 response is characterized by the production of IFN-γ and TNF by T cells. It is the normal outcome after an exposure of DCs to viruses or bacteria. It is also the basis of the delayed type hypersensitivity reaction. Th2 differentiation usually occurs following contact with extracellular parasites and involves the production of cytokines IL-4, IL-5, IL-9, and IL-13 resulting in IgE production and accumulation of eosinophils and mast cells. Furthermore, in allergic asthma, as nonpathogenic environmental antigens are able to induce an inappropriate Th2 response and become allergens, such as the house dust mite allergen Der p1. The third outcome is the induction of regulatory T cells that produce immunosuppressive cytokines such as IL-10 or TGF-β. This describes probably the most prevalent response in steady-state conditions, as it forms a constant safeguard against the induction of inappropriate inflammatory reactions to harmless antigen [37]. It has become increasingly evident that T cell functions are considerably more complex and heterogeneous than originally assumed. In particular, the potential key role of Th17 cells in disease pathogenesis has been described. As an example, some asthma patients have been described to develop a more type 17 associated disease with dominance of neutrophils rather than eosinophils [38]. An additional conceptual development has emerged with the role of airway epithelial cells in driving the selection of disease-related T cell phenotypes through the expression of potent T cell modulatory molecules (discussed in [19]).

T cells are also found in varying numbers in the airways and the lung parenchyma. In the airways they are found intraepithelially and within the underlying lamina propria. As in the gut, most intraepithelial T cells express CD8, whereas CD4^+^ T cells are more frequently localized the lamina propria. Both subsets mainly have an effector- and/or memory-cell phenotype [19]. Both in vitro and in vivo studies have shown that T cell proliferation upon NP treatment can be affected [8, 22, 39–41]. T cells are thus promising targets for future therapies using biomedical NPs.

**Fig. 2:**
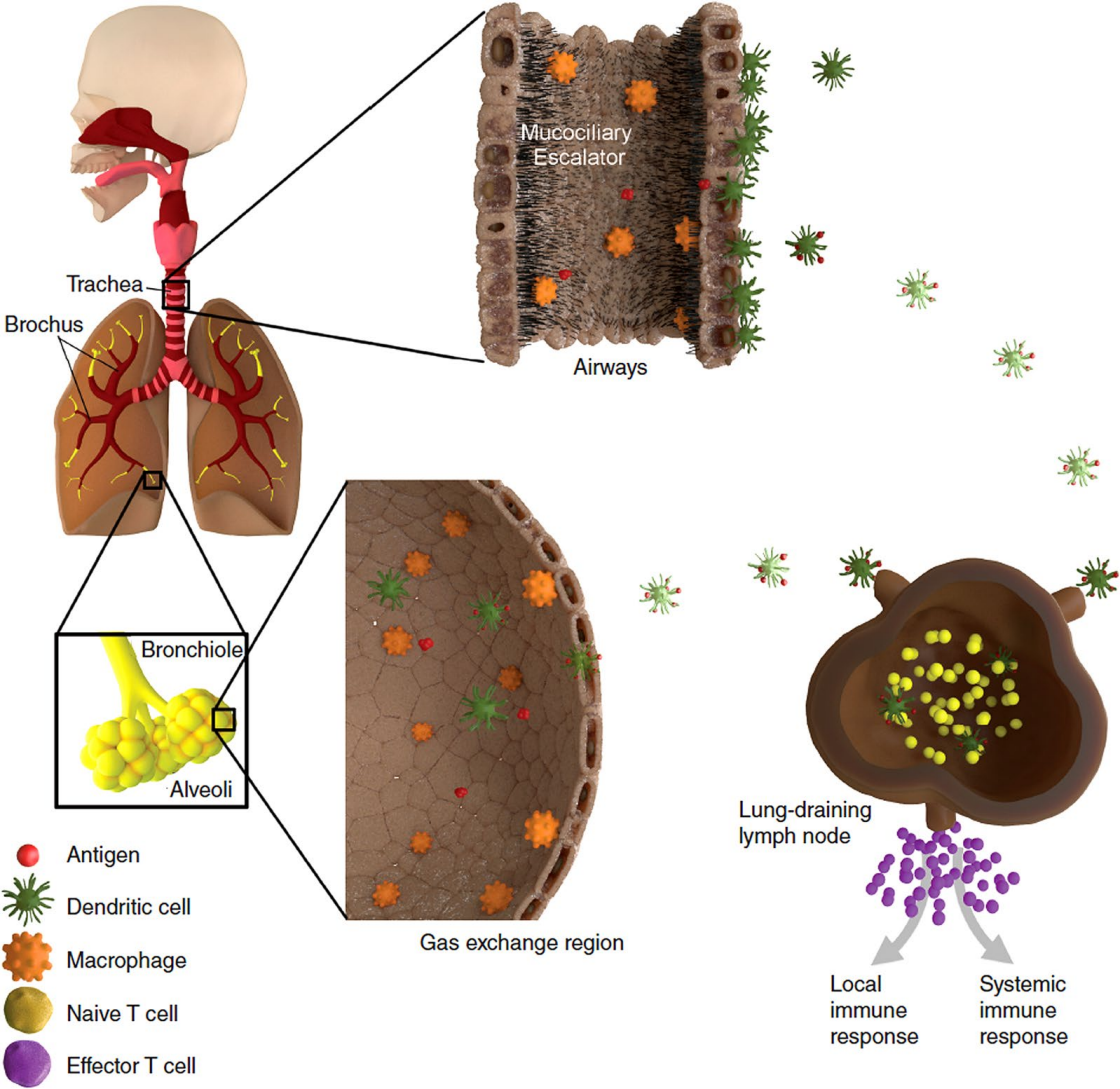
Simplified schematic presentation of the human respiratory immune system. The upper respiratory epithelium, lining the inner surface of the trachea, bronchi and bronchioles, is composed of a pseudostratified layer of ciliated cells, mucus-producing cells and basal cells, and is responsible for rapid clearance of inhaled particulate antigen with the mucociliary escalator. The distal regions of the lung epithelium, the alveolar septa, represent the site of the gas exchange. In both regions, macrophages are located at the apical side of the epithelial layer and protect it from the inhaled antigen cells by phagocytosis. Dendritic cells will capture antigens, process and present antigen peptide to naive T cells, and trigger their differentiation into antigen-specific effector T cells. Figure as shown in and reprinted with permission from *Nanomedicine* (*Futuremedicine*)

The lamina propria of the airways also contains mast cells and plasma cells (mainly producing polymeric IgA) and some loosely distributed B cells. Aside from their central role in antibody production, it is possible that B cells also contribute to local antigen presentation, given the recent demonstration of such a function for B cells in the lymph nodes that drain the lungs [42]. Figure 2 shows a simplified illustration of the innate and adaptive immune response in the respiratory tract.

## Particle deposition in different lung compartments

According to the particle size, it can be predicted in which compartment particles will be predominantly deposit in the lung [10, 43]. Larger particles (1–10 μm) preferentially deposit in trachea and bronchi, whereas smaller particles (i.e. NPs) tend to deposit in in the deeper regions of the lung (i.e. small airways and alveoli). Inhaled particles may be deposited in the lung by impaction, sedimentation and diffusion as described in detail in [44]. While impaction is generally observed with particles greater than 5 μm, sedimentation is seen with particles with sufficient mass and a size of 1–5 μm in diameter. Finally diffusion is observed mainly with the smallest particles (Table 1). Therefore, solely depending on the size of particles or aerosol droplets, different compartments of the respiratory tract and specific subpopulations of immune cells may be targeted. In addition a recent study has shown that depending on size and charge particles deposited on the respiratory mucus are either locally trapped or can diffuse freely [45].

**Table 1:** Correlation between compartments of lung deposition, the mechanism of deposition and particle size

Location	Size (μm)	Mechanism
Primary bronchi	5–10	Impaction
Secondary bronchi	1–5	Sedimentation
Bronchioles	1–3	Sedimentation
Alveoli	0.5–1	Brownian motion

A large number of different studies in the recent years has also demonstrated that characteristics of NPs like size, shape, surface charge, and surface modification all play an important role in affecting the fate of the particles in the respiratory tract with particle size, surface charge and surface modification being among the most important. Deposition in the respiratory tract depends, however, mainly on particle size due to the fact that different mechanisms of particle deposition are defined based on this characteristic.

Based on size-dependent pulmonary deposition NPs can be used to primarily target distal lung compartments for prolonged persistence, since in these anatomical areas there is only slow removal by alveolar macrophages compared to the proximal lung compartments like the conducting airways. Prolonged persistence allows NPs to interact with cells of interest for a longer time in order to become effective locally by remaining in the lung compartment or systemically by crossing the air-blood barrier. In particular the interaction of NPs with pulmonary immune cells is of great interest, since NPs can be easily applied in the lungs and immediately get in contact with different cells of the immune system after deposition. A number of recent studies has characterized how inhaled NPs affect immune cells in the lung and provide valuable information for the development of novel biomedical tools for pulmonary delivery.

## Immunomodulatory potential of NPs in in vivo models

The highly complex organization of the pulmonary immune system characterised by a multitude of cell–cell interactions across different respiratory tract compartments, highlights the essential requirement to investigate the fate and effects of inhalable biomedical NPs. Hence in vivo models are a crucial step in the optimization of potential biomedical NPs following initial development through in vitro investigations [13] before clinical studies can be considered. In the following paragraph promising nanocarriers and treatment strategies, which have been tested in in vivo models, i.e. mainly rodents, are discussed and compared.

Screening for NP characteristics relevant for translocation in the respiratory tract, Choi et al. utilized different NPs by varying material, size, shape, as well as surface charge, and correlated these properties with translocation in the body and adverse health effects, after lung instillation in rat models [46]. Briefly, administration of non-cationic NPs with a size of approximately 30 nm or smaller resulted in a maximal translocation to mediastinal lymph nodes and the bloodstream due to insufficient clearance. The authors suggested to employ chemical modifications to adapt size and the charge of NPs, so the adverse health effects may be minimized. Focusing on particle size, we employed in a recently reported in vivo study polystyrene (PS) NPs intra-nasally in mice, and demonstrated size-dependent uptake, trafficking, and modulation of downstream immune responses [22]. Compared to larger NPs, those with a diameter of 20–50 nm were preferentially captured and trafficked by pulmonary DCs to lung draining lymph nodes, while very low or no lymphatic drainage was observed with any other particle size. In particular, 20 nm PS NPs co-administered together with the model antigen ovalbumin (OVA) induced significantly enhanced activation of antigen-specific T cells, compared to results obtained with larger 1000 nm particles [22]. In contrast, a similar study done by Hardy and co-workers showed a prophylactic inhibitory effect of 50 nm neutral amino acid glycine (PS50G) NPs: Intratracheally instilled PS50G NPs did not exacerbate but instead inhibited key features of allergic airway inflammation including lung airway and parenchymal inflammation, airway epithelial mucus production, and serum allergen-specific IgE and allergen-specific Th2 cytokines in the lung-draining lymph node after allergen challenge 1 month later. Furthermore, PS50G NPs themselves did not induce any inflammatory response or oxidative stress in the lungs. Finally, PS50G NPs suppressed the ability of CD11b^hi^ DCs in the draining lymph nodes of allergen-challenged mice to induce proliferation of OVA-specific CD4^+^ T cells [41]. A follow-up study of the same group using the same PS50G (50 nm) and larger PS500G (500 nm) nanoparticles, investigated the uptake by antigen presenting cell populations in the lung parenchyma and the lung draining lymph nodes following intra-tracheal instillation in naive mice. It was found that PS50G were preferentially taken up by alveolar and non-alveolar macrophages, B cells, and CD11b^+^ and CD103^+^ DC in the lung. However, in the lung draining lymph nodes, only DCs were found to contain particles, demonstrating transport of NPs to the lymph nodes exlusively by DCs. Consisten with our findings, this study excluded particle translocation via lymphatic drainage. However, both particle sizes decreased frequencies of stimulatory allergen-laden DC in the lung draining lymph nodes, with the smaller particles having the more pronounced effect. The authors from these studies concluded that in allergic airway inflammation PS50G but not PS500G significantly inhibited adaptive allergen-specific immunity [47]. Another study with results similar to our findings investigated the trafficking of intranasal instilled 500 nm PS beads from the respiratory tract to the mediastinal lymph nodes, in which the majority of particles was captured by alveolar macrophages, but particles were also detected in a small number of DCs that had migrated to the T cell—rich areas of the mediastinal lymph nodes [23]. Additional studies have shown that polylactid-co-Glycolid (PLGA) NPs (approximately 200 nm) and dendrimers (<10 nm) may be functionalized with siRNA or drugs while surface charge can be controlled during synthesis of NPs [48–50]. Focusing on pulmonary deposition following inhalation, Taratula et al. [51] successfully delivered a high concentration of inhalable lipid-NP-based drug to the respiratory tract of mice. In this study, pulmonary deposition was more efficient compared to intravenous injection of the same drug, in terms of organ distribution, lung tumor targeting, and anti-cancer activity. These studies demonstrate a significant effect of particle size in the modulation of innate and adaptive immune responses in the respiratory tract. Particle size has therefore to be taken in consideration for the development of biomedical carriers for the use in pulmonary applications.

**Fig. 3:**
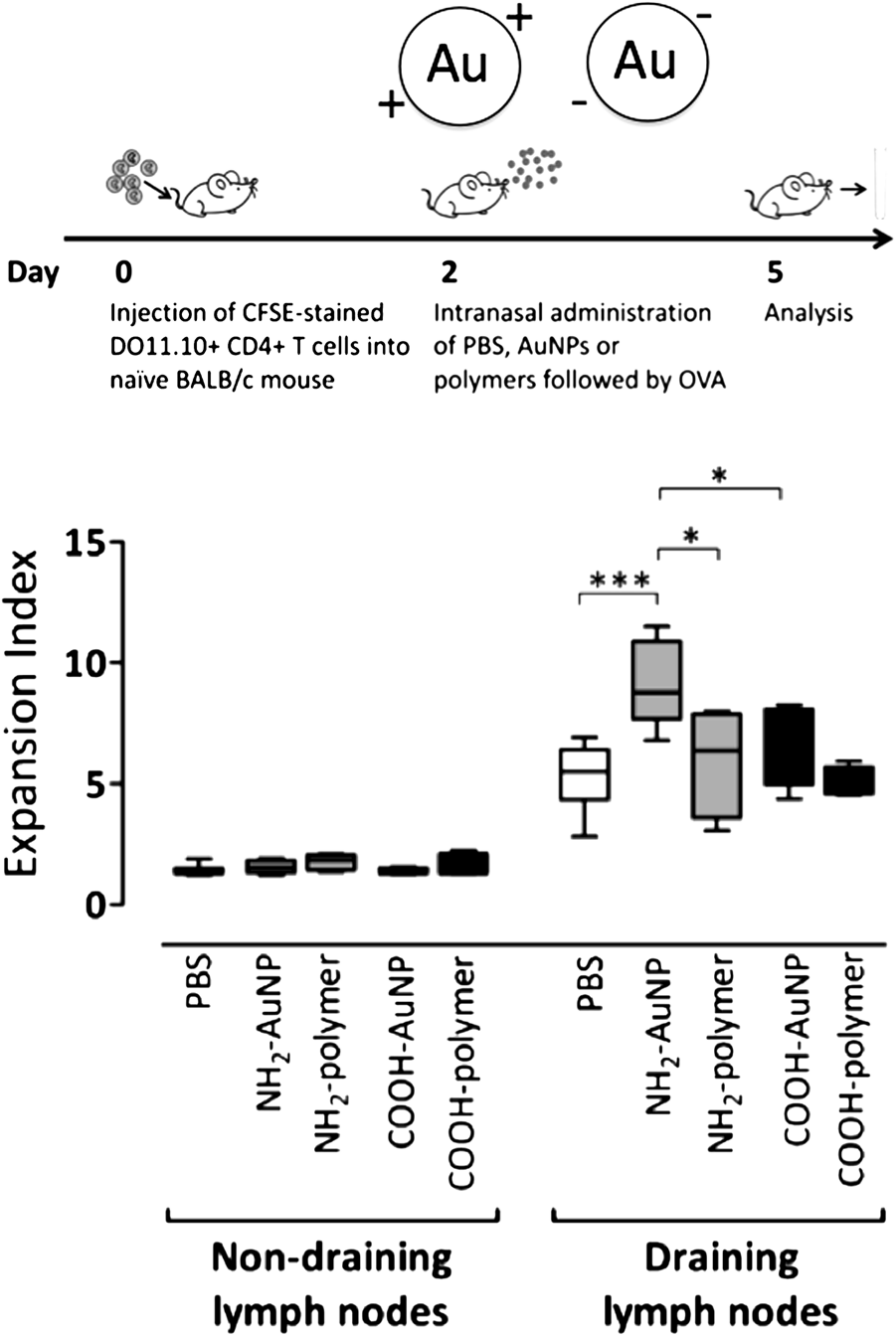
CD4^+^ T cell proliferation in lung draining lymph nodes was measured after intra nasal instillation of positively charged (Au^+^; NH2) and negatively charged (Au^−^; COOH) gold NPs or polymer shells alone followed by ovalbumin in a mouse model of ovalbumin induced experimental allergic airways disease. Positively charged gold NPs induced enhanced ovalbumin specific T cell proliferation compared to controls (non-exposed), negatively charged gold NPs or positively charged polymer alone. These findings highlight the importance of surface charge of a biomedical NP in modulating a specific adaptive immune response. Adapted from [11]

As already discussed above, not only particle size but also surface charge of an engineered NP may affect pulmonary immune cells and modulate downstream immune responses. In order to address how surface charge of a pulmonary administered NP may affect its fate and modulate a specific immune response, we employed modified gold NPs (AuNPs) (Fig. 3). The AuNPs were coated with polyvinyl alcohol (PVA) containing either positively (NH_2_) or negatively (COOH) charged functional groups [52]. Following intra-nasal instillation in a mouse model, all pulmonary APC subsets preferentially took up positively charged AuNPs, compared to negatively charged AuNPs. Also, positively charged AuNPs generated an enhanced ovalbumin-specific CD4^+^ T cell stimulation in lung draining lymph nodes compared to negatively charged AuNPs. An additional salient finding in this study was that intact positively charged AuNPs were necessary, as immune responses were not affected when the positively charged polymer was utilized alone. Another recent study also demonstrated improved therapeutic effects of a particulate biomedical carrier compared to its soluble counterpart: In this study solid lipid nanoparticles (SLNs) of Yuxingcao essential oil (YEO) with different particle size (200, 400 and 800 nm) were prepared using Compritol 888 ATO as lipid and polyvinyl alcohol as an emulsifier. Following intra-tracheal administration in rats, YEO loaded SLNs not only prolonged pulmonary retention up to 24 h, but also increased area under the curve values (15.4, 18.2 and 26.3 μg/g h for SLN-200, SLN-400 and SLN-800, respectively) by 4.5–7.7 folds compared to the intra-tracheal dosed YEO solution and by 257–438 folds to the intravenously dosed YEO solution, respectively. These results demonstrated a promising inhalable particulate carrier with improved local bioavailability [53]. Furthermore, a similar study showing effects of surface charge following administration to the lung was conducted to understand the biological impact of superparamagnetic iron oxide NPs (SPIONs) and their surface-modification with polyethylene glycol having either negative (i.e. carboxyl) or positive (i.e. amine) functional groups in a 1-month longitudinal study using a mouse model. Genetic assessment revealed enhanced expression of chemokine ligand 17 (CCL-17) and IL-10 biomarkers following SPIONs administration compared to surface-modified NPs. However, SPIONs with carboxyl terminal showed a slightly prominent effect compared to amine modification [54]. A further study used cationic carbon dots for pulmonary delivery of DNA. Administration of particle-DNA complexes to mouse lungs demonstrated that these new carriers achieved similar efficiency but lower toxicity compared to GL67A, a golden standard lipid based transfection reagent for gene delivery to the lungs. The authors suggested that post-functionalization of these nanoparticles with polyethylene glycol (PEG) or targeting moieties should even improve their efficiency and in vivo biocompatibility [55]. Another recent in vivo study performed with hydrogel rod-shaped NPs of different surface charge also confirmed enhanced uptake of positively charged NPs by alveolar macrophages and different subsets of pulmonary DC, with enhanced trafficking to lung draining lymph nodes, as compared to negatively charged NPs. The authors concluded that cationic NPs are endowed with an enhanced immunomodulatory potential in the respiratory tract [56]. All these in vivo findings underline that size, surface charge and intact conformation of engineered NPs play an important role in modulating downstream immune responses in the respiratory tract [11]. The studies discussed above highlight that different attributes of NPs such as size and surface charge may become important triggers to re-program adaptive immune responses in the respiratory tract. Inhalable NPs may therefore be designed to specifically modulate pulmonary immune responses, either towards an immune-therapy to reprogram allergic responses, or vaccination to generate protective immunity against a respiratory pathogen. To prevent triggering of excessive inflammatory responses that may jeopardise gaseous exchange, meticulous development of inhalable NPs through in depth characterisation of in vivo effects is the final, but most crucial step in pre-clinical development.

## Conclusions

The lung with its extensive internal surface harboring different immune cell populations, provides a non-invasive and promising target organ for novel therapies with inhalable nanoparticles. NP engineering approaches is a promising technique for non-invasive and cost-effective pulmonary drug delivery to treat respiratory tract disorder. Specific NP properties such as material, size and surface modification that can be used to stimulate or to inhibit a specific immune reaction may be specifically designed for the treatment of immune disease, such as allergic asthma. In order to achieve this, close collaboration and interdisciplinary research between physicians, biologists, chemists and material scientists is essential. Furthermore, careful design, thorough characterization and process control in the entire NP synthesis procedure is required in order to assure high-quality NP batches with repeatedly reproducible and accurate results. Sophisticated approaches using relevant animal models can play a major role in this development since they can provide straight-forward and reliable data which can be the baseline of such developments.
